# Supplementary material for: Representation of Parkinson's disease and atypical Parkinson's syndromes in the Czech Republic—A nationwide retrospective study
Source: PLoS One. 2021 Feb 2;16(2):e0246342. doi: 10.1371/journal.pone.0246342 (PMC7853522; doi:10.1371/journal.pone.0246342)
Supplement: Attachment — Submitted filename: Response to Reviewers_6_1_2021.docx [file pone.0246342.s002.docx]

**Response to Reviewers**

**PONE-D-20-31004**

**Representation of Parkinson's disease and atypical Parkinson's syndromes in the Czech Republic - A nationwide retrospective study**

The authors would like to thank the reviewers for their suggestions, comments and advices which helped them to improve
the manuscript. We did our best to address all the comments and make appropriate changes in the manuscript.

| **Reminders** | **Reviewer´s** | **Reaction of the author's team** |
| --- | --- | --- |
| **Reviewer #1:** If the authors have adequately addressed your comments raised in a previous round of review and you feel that this manuscript is now acceptable for publication, you may indicate that here to bypass the “Comments to the Author” section, enter your conflict of interest statement in the “Confidential to Editor” section, and submit your "Accept" recommendation. | | |
| In the abstract and in the manuscript: in the aim of the study, " The demographic description of reported patients with Parkinson's disease"==> The demographic description of reported patients with parkinsonism (including Parkinson's disease and atypical parkinsonian syndromes): the aim should be in accordance with the article title. | **Reviewer #1** | Accepted – the aim has been reworded according the reviewer´s comment. |
| The authors should precise all the data analyzed later in the results in the methodology part. This was not amended. | **Reviewer #1** | Accepted – the information in the methodology part has been added. |
| The quality of figures and tables should be improved. | **Reviewer #1** | Accepted – the figures were graphically edited and uploaded. |
